# ‘The kind of doctor I wanted to be.’ A qualitative analysis of junior doctors’ reasons for choosing to train in psychiatry and in Wales

**DOI:** 10.1192/bjo.2021.919

**Published:** 2021-06-18

**Authors:** Alan Slater, Rugi Saeed, Marque Fernando, Ceri Evans

**Affiliations:** 1 Cardiff and Vale University Health Board; 2 Cwm Taf University Health Board

## Abstract

**Aims:**

To understand the factors underpinning junior doctors’ decision-making processes regarding their choice of psychiatry as a speciality, and why they chose to pursue specialty training in Wales.

**Background:**

Over recent years there have been significant challenges in recruiting junior doctors into psychiatry specialty training, both within the UK and in Wales. To counter this a number of measures have been instituted, including advertising campaigns from the Royal College of Psychiatrists (‘Choose Psychiatry’) and Health Education and Improvement Wales (HEIW) (‘Train Work Live’), together with financial incentives. To date there has been no published evaluation of the effectiveness of these measures.

**Method:**

Two focus groups were run (in August 2019 and January 2020) with trainees appointed to new training posts in August 2019. The focus groups featured set questions acting as prompts for discussion. These focused on various factors behind making decisions to train in Psychiatry and choosing to train within HEIW. The focus groups were recorded and transcribed. Following this a thematic analysis was conducted to identify key elements arising from the discussions.

**Result:**

The focus groups were attended by 14 trainees in total (eleven CT1s, four ST4s.) Living in Wales prior to appointment was the most common factor in leading participants to choose to train in Wales, jointly with having a support network (friends or family) in Wales (each present in 57%, n = 8.) Perceptions around a favourable work-life balance were also important (45%, n = 5.) Interactions with staff in an ambassadorial or support role were a significant driver, especially for international medical graduates. Financial incentives and advertising campaigns appeared to have limited influence over participants’ decision-making, awareness of these being highest among those already working in psychiatry or in Wales.

Having a foundation year job with a psychiatry placement was a common theme in choosing psychiatry as a specialty (43%, n = 6.) Work-life balance of the specialty was also important (21%, n = 3.) Again, after these it was hard to identify coherent themes.

**Conclusion:**

We identified three separate groups, namely CT1s, ST4s and international medical graduates, each with distinct themes underlying a range of needs. There was a broad range of factors underlying trainees’ decisions which should be reflected when planning future recruitment strategies. It appeared that advertising campaigns and financial incentives were of limited influence.

